# Vitamin D Receptor Gene Polymorphisms on the Risk of Tuberculosis, a Meta-Analysis of 29 Case-Control Studies

**DOI:** 10.1371/journal.pone.0083843

**Published:** 2013-12-13

**Authors:** Cheng Chen, Qiao Liu, Limei Zhu, Haitao Yang, Wei Lu

**Affiliations:** Department of Chronic Communicable Disease, Center for Disease Control and Prevention of Jiangsu Province, Nanjing, China; National Institute for Public Health and the Environment, Netherlands

## Abstract

The relationship of four potentially functional polymorphisms of the vitamin D receptor (VDR) gene, ApaI, BsmI, FokI and TaqI , with tuberculosis susceptibility were considered. The aim of this meta-analysis was to explore the association between the four polymorphisms and tuberculosis risk in different ethnic backgrounds. Eligible case-control studies that were catalogued before April 1^st^ 2013 were enrolled, and the heterogeneity between the studies was evaluated using a χ^2^ based *Q*-test. Fixed and random effect models were built to evaluate the association of the four polymorphisms with the risk of tuberculosis, and the association between the four polymorphisms and tuberculosis was expressed as the odds ratio (OR) and 95% confidence interval (CI). Finally, twenty nine qualified studies were enrolled for this meta-analysis that included 6179 tuberculosis cases and 6585 healthy controls. The variant homozygote genotype of the FokI polymorphism was associated with a significantly increased risk of tuberculosis when compared to the heterozygote and wild type homozygote genotypes in the Chinese population (ff vs. Ff+FF: OR_*recessive*_=1.97, 95%CI: 1.32-2.93, *P*_*bonferroni*_=0.0032; heterogeneity test: χ^2^=0.24, *P*=0.62). For European subjects, the homozygote and heterozygote genotypes of the BsmI polymorphism were associated with a significantly decreased risk of tuberculosis when compared to the wild type homozygote (bb+Bb vs. BB: OR_*dominant*_=0.41, 95%CI, 0.22-0.76, *P*_*bonferroni*_=0.02; heterogeneity test: χ^2^=2.59, *P*=0.11). Based on the above results, we conclude that variants of the VDR gene that are homozygous for the FokI polymorphism might be more susceptible to tuberculosis in Chinese. Furthermore, larger sample studies are warranted to confirm the protective effects of BsmI variants on tuberculosis in the Europeans.

## Introduction

Several studies have revealed that a deficiency of vitamin D would increase the risk of tuberculosis [1,2], and vitamin D from food or exposure to sunlight would benefit the treatment of tuberculosis [3-5]. 1,25-dihydroxyvitamin D_3_, which is the active form of vitamin D, was activated by 1α-hydoxylase that was expressed by macrophages and other immune cells and bind to the vitamin D receptor (VDR) for to modulate the immune system in fighting with *Mycobacterium* infection [6]. 

In recent years, the anti-bacteria effects of Vitamin D in the human immune system have been explored extensively [7]. Meanwhile, host-genetic susceptibility was considered contribute to the risk of tuberculosis development. The VDR gene is located in the chromosomal 12q13 region, and, in the last decade, studies have focused on the association between genetic variants of the VDR gene and tuberculosis risk based on the hypothesis that genetic alterations of the VDR gene might lead to important defects in gene activation, that could affect immune function. For instance, deleterious mutations in the VDR gene cause 1, -25-dihydroxivitamin D resistant rickets, a rare monogenetic disease [8]. Four potentially functional single nucleotide polymorphisms (SNPs) of the VDR gene (ApaI, SNP ID: rs7975232; BsmI, SNP ID: rs1544410; FokI, SNP ID: rs2228570; and TaqI, SNP ID: rs731236) have been main focus in relation to the risk of tuberculosis compared to other SNPs. Changes in the amino acid sequence or transcriptional activity might be induced by these four mutations; therefore, these mutations could affect an individual’s susceptibility to tuberculosis.

Due to underpowered design or small study sample size, previous studies have not reached a consensus regarding the association between the VDR gene variants and tuberculosis risk [9-11]. A previous meta-analysis conducted by Gao et al reported marginal, significant associations of the ApaI and TaqI polymorphisms with tuberculosis risk [12], but this finding needs to be validated in a larger pooled sample size. After the results reported by Gao et al, nearly ten research studies were conducted to elucidate the relationship between the VDR gene polymorphisms and tuberculosis risk, and their results were also inconsistent. Additionally, studies of European ethnicity [13,14] were not included in the analysis in the previous meta-report. Thus, because previous results were inconsistent and more ethnic background needed to be added, it was necessary to carry out this meta-analysis on the relationship between hot spot variants of the VDR gene and the risk of tuberculosis.

## Methods

### Search protocol and eligibility of enrolled studies

We searched two databases for association studies of VDR gene polymorphisms and tuberculosis: PubMed and Web of Science. We conducted the search from March 18^th^ 2013 to April 1^st^ 2013, and all the searched results were finally updated on April 1^st^ 2013. All articles catalogued before April 1^st^ 2013 were considered. Two medical subject headings (Mesh), “Tuberculosis” and “Vitamin D receptor”, were used to search for related articles, and qualified articles were included according to the following criteria: (1) the original articles were published in English; (2) the studies had a case-control design and were conducted in human beings; and (3) the articles contained available genotyping information on VDR gene polymorphisms (ApaI, BsmI, FokI and TaqI were the four SNPs that were analyzed in this article). Two independent investigators were employed to search the related articles according to the above criteria.

### Data extraction

The two investigators independently extracted data and reached a consensus on all of the items. If the two investigators generated different results, they would check the data again and finally reach a consensus. An expert would be invited to the discussion if they could not reach an agreement. Data extracted from the selected articles included the first author’s name, year of publication, country of origin, ethnicity, number of cases and controls, diagnosis method of cases and the selection of controls. 

### Statistical analysis

The association between the four polymorphisms and risk of tuberculosis for each study was estimated by the odds ratio (OR) and 95% confidence interval (CI). A χ^2^-test-based *Q* statistic test was performed to assess the between study heterogeneity, and *P*<0.05 was considered a significant level. A fixed-effect model by the Mantel–Haenszel method and a random-effect model by the DerSimonian and Laird method were adopted for combining data. When the P value of heterogeneity was <0.05, then a random-effect model was employed. Otherwise, the fixed-effect model was adopted throughout the analyses. These two models provided similar results when heterogeneity between studies was absent; otherwise, a random-effect model was more appropriate. Bonferroni correction was adopted for multiple comparisons.

Heterozygote and variant homozygote of each SNP was compared with the wild-type homozygote to estimate the risk of tuberculosis. Then, a dominant model, that combined variant homozygote and heterozygote genotypes, was established to calculate the risk of tuberculosis, versus the wild-type homozygote. Meanwhile, heterozygote and wild-type homozygote genotypes of each SNP were also combined as a recessive model to evaluate the risk of tuberculosis, versus the variant homozygote.

Stratification analyses were performed based on ethnicity and region, and a meta regression was used to illustrate the potential reasons for heterogeneity between the studies. Inverted funnel plots, an Egger’s test and a Horbold-Egger’s test were used to assess publication bias [15]. Meta-analysis was carried out using Review Manager (V5.2, The Cochrane Collaboration, Oxford, UK); meanwhile, Egger’s test for publication bias was conducted using Stata software (V10.0, Stata, College Station, TX, USA), and evaluations were made assuming a two-sided test with a significance level of 0.05.

## Results

### Characteristics of enrolled studies

**Figure 1:**
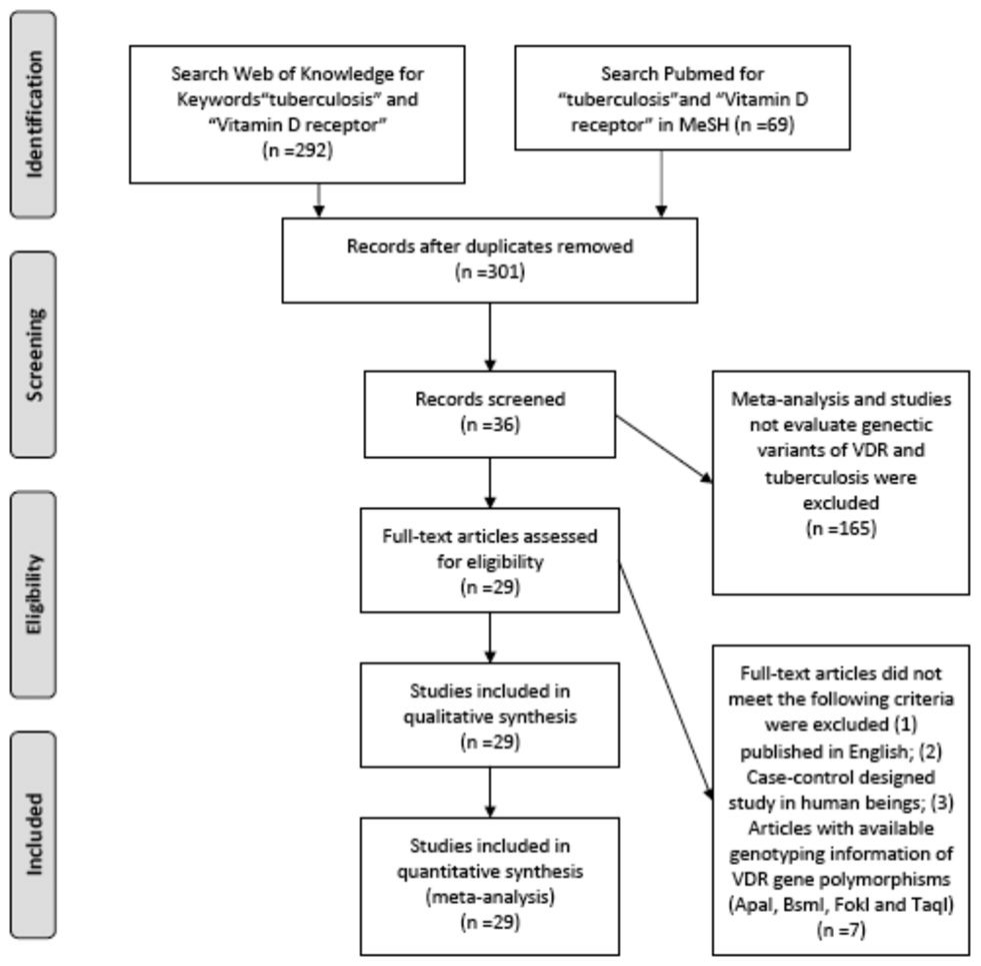
Flow chart of study inclusion.

A flow chart of the inclusion and exclusion procedures of the articles is shown in Figure 1. According to the inclusion criteria, twenty nine qualified case-control studies were included in the final analysis [10,11,13,14,16-40]. Seventeen studies were conducted in Asia, seven in Africa and the remaining five were conducted in Europe and America. The basic information of the enrolled studies was listed in Table 1. A total of 6179 tuberculosis cases were obtained in the twenty nine studies, including 5306 (85.9%) with pulmonary tuberculosis, 383 (6.2%) with extra pulmonary tuberculosis, and 490 with (7.9%) tuberculosis without a specific disease origination part of the body. The corresponding controls for the tuberculosis cases were 6585. Meanwhile, the HIV (Human Immunodeficiency Virus) status was not available in eleven (37.9%) studies. In this meta-analysis, SNPs (ApaI and BsmI) near the 3′ untranslated region (UTR) and SNPs (FokI and TaqI) in the coding region were evaluated. The TaqI polymorphism was the most evaluated SNP in the twenty five case-control studies, while FokI was found in twenty three studies, BsmI was found in eighteen studies and ApaI was found in fifteen studies.

**Table 1:** Basic information of qualified cases-control studies on vitamin D receptor gene polymorphisms for tuberculosis.

Year	First Author	Country	Ethnicity	Tuberculosis			Controls		HIV status	SNPs
				Part of the body	Sample size	Diagnosis method		Sample size		
1999	Bellamy	Gambia	African	Pulmonary tuberculosis	408	AFB smear	Male donors	414	All tested negative	TaqI
2000	Selvaraj	India	Asian	Spinal tuberculosis	66	Culture and X-ray	Spouses of TB contacts	80	Not available	TaqI
2000	Wilkinson	India	Asian	Pulmonary tuberculosis(27) and military tuberculosis (64)	91	Biopsy or culture	Tuberculosis contacts with no TB	116	All tested negative	FokI, TaqI
2002	Delgado	Cambodia	Asian	Pulmonary tuberculosis	358	AFB smear	Control with no signs or symptoms of TB	106	All tested negative	TaqI
2002	Fibla	Spain	European	Pulmonary tuberculosis(48) and extra pulmonary tuberculosis (18)	66	Culture and X-ray	Healthy persons	136	Cases positive, Controls negative	BsmI
2003	Selvaraj	India	Asian	Pulmonary tuberculosis	120	Culture	patient contacts	80	Not available	FokI, ApaI, BsmI
2004	Bornman	Gambia, Guinea-Bissau, Guinea	African	Pulmonary tuberculosis	417	AFB or culture	healthy community control subjects	722	Cases (12.5%),controls(6.8%)	FokI, ApaI, TaqI, BsmI
2004	Fitness	Malawi	African	Tuberculosis	386	AFB smear, culture and histology	age, sex and area of residence matched with cases	624	HIV positive in 67.6% cases and 13.1% controls	ApaI, TaqI, BsmI
2004	Liu	China	Asian	Pulmonary tuberculosis	120	AFB smear, culture and X-ray	normal controls	240	All reported negative	FokI, TaqI
2004	Roth	Peru	American	Pulmonary tuberculosis	103	AFB smear	Two healthy controls,1PPD+ and 1PPD-	206	All reported negative	FokI, TaqI
2004	Selvaraj	India	Asian	Spinal tuberculosis patients	64	X-ray and Clinical criteria	Normal healthy controls	103	Not available	FokI, ApaI, TaqI, BsmI
2006	Lombard	Venda	African	Pulmonary and meningeal tuberculosis	104	AFB smear	Healthy controls with no history of TB	117	All reported negative	FokI, ApaI, TaqI, BsmI
2007	Babb	South Africa	African	Pulmonary tuberculosis	249	AFB smear and X-Ray	No clinical history or symptoms of TB	352	All reported negative	FokI, ApaI, TaqI
2007	Olesen	Guinea-Bissau	African	Pulmonary tuberculosis	321	AFB smear and clinical critiera	Healthy controls	347	HIV positive in 33% cases and negative in controls	FokI, ApaI, TaqI, BsmI
2007	Soborg	Tanzanian	African	Pulmonary tuberculosis	443	Culture	Culture negative	426	HIV positive in 44% cases and 18% controls	FokI, ApaI, TaqI
2007	Wilbur	Paraguay	American	Pulmonary tuberculosis	54	Clinical symptoms, PPD test	No symptoms	124	Not available	FokI, TaqI
2008	Selvaraj	India	Asian	Pulmonary tuberculosis	51	AFB smear and culture	Normal healthy subjects	60	All reported negative	FokI, ApaI, TaqI, BsmI
2009	Alagarasu	India	Asian	Pulmonary (190) and extra pulmonary tuberculosis (31)	221	AFB smear, clinical criteria and X-ray	Healthy controls	146	Cases(51%),Controls(0)	FokI, ApaI, TaqI, BsmI
2009	Merza	Iran	Asian	Pulmonary Tuberculosis	117	AFB smear and X-ray	Healthy individuals	60	Not available	FokI, BsmI
2009	Selvaraj	India	Asian	Pulmonary tuberculosis	65	Clinical symptom, AFB smear and culture	Healthy subjects	60	All reported negative	FokI, ApaI, TaqI, BsmI
2009	Vidyarani	India	Asian	Pulmonary tuberculosis	40	AFB smear and culture	Normal healthy subjects	49	Not available	FokI, ApaI, TaqI, BsmI
2010	Banoei	Iran	Asian	Pulmonary tuberculosis	60	Confirmed in Massih Daneshvari Hospital	Healthy subjects	62	All reported negative	FokI, TaqI, BsmI
2010	Marashian	Iran	Asian	Pulmonary tuberculosis	164	AFB smear and X-ray	Healthy People	50	Not available	FokI, ApaI, TaqI, BsmI
2010	Zhang	China	Asian	Spinal tuberculosis	110	Postoperative pathology	Healthy subjects	102	All reported negative	FokI
2011	Ates	Turkey	European	Pulmonary (98) and extra pulmonary tuberculosis (30)	128	AFB and Culture	Healthy subjects	80	Not available	FokI, TaqI, BsmI
2011	Sharma	India	Asian	Pulmonary tuberculosis	992	AFB smear and Culture	Healthy subjects	1183	Not available	FokI, ApaI, TaqI, BsmI
2011	Singh	India	Asian	Pulmonary tuberculosis	101	AFB smear or culture	healthy controls	225	All reported negative	FokI, TaqI, BsmI
2012	Rathored	India	Asian	MDR tuberculosis and drug sensitive pulmonary tuberculosis	692	AFB smear and culture	healthy volunteers	205	Not available	FokI, TaqI, BsmI
2013	Alexandra	Romania	European	Pulmonary tuberculosis	68	Not available	unrelated healthy individuals	110	Not available	ApaI, TaqI

^a^ AFB, Acid-fast bacilli; HIV, human immunodeficiency virus; MDR, multi-drug resistance for isoniazide and rifampicin; PPD, purified protein derivative; SNPs, single nucleotide polymorphism; TB, tuberculosis.

### Quantitative synthesis

In Table 2, four SNPs and their relationship to the risk of tuberculosis were evaluated by four types of comparisons: (1), heterozygote genotype versus wild-type homozygote genotype; (2), variant homozygote genotype versus wild-type homozygote genotype; (3), dominant model: variant homozygote genotype combined with a heterozygote genotype versus wild-type homozygote genotype; and (4), recessive model: variant homozygote genotype versus heterozygote and wild-type homozygote genotypes). As shown in Table 2, when the P value of the heterogeneity test between the studies was less than 0.05, a random-effect model was used to calculate the odds ratios and 95% confidence interval. Otherwise, a fixed-effect model was adopted. 

**Table 2:** Summary of VDR gene genotypes on the risk of tuberculosis in different ethnicities.

SNP	Ethnicity	Cases			Controls			HT vs. WT Homo	^e^ *P*-value	VR homo vs. WT Homo	^e^ *P*-value	Dominant model^a^	^e^ *P*-value	Recessive model^b^	^e^ *P*-value
		WT Homo n(%)	HT n(%)	VR Homo n(%)	WT Homo n(%)	HT n(%)	VR Homo n(%)	OR(95%CI)		OR(95%CI)		OR(95%CI)		OR(95%CI)	
ApaI		AA	Aa	aa	AA	Aa	aa								
	Total	**1028**	**1423**	**540**	**1265**	**1720**	**646**	1.00 [0.84, 1.20] ^c^	1	0.88 [0.65, 1.19] ^c^	1	0.97 [0.81, 1.16] ^c^	1	0.89 [0.78, 1.02]	0.4
	ES Asians	0	0	0	0	0	0	NA		NA		NA		NA	
	SW Asians	461	530	137	425	567	120	0.91 [0.67, 1.25] ^c^	1	0.94 [0.71, 1.26]	1	0.92 [0.69, 1.24] ^c^	1	1.01 [0.77, 1.32]	1
	Africans	548	844	403	813	1095	501	1.06 [0.82, 1.36] ^c^	1	0.92 [0.64, 1.32] ^c^	1	1.03 [0.79, 1.34] ^c^	1	0.91 [0.77, 1.07]	0.92
	Americans	0	0	0	0	0	0	NA		NA		NA		NA	
	Europeans	19	49	0	27	58	25	1.20 [0.60, 2.42]^d^	1	0.00 [0.00, 0.06]^d^	0.0004	0.84 [0.42, 1.66]^d^	1	0.02 [0.00, 0.41]^d^	0.04
BsmI		BB	Bb	bb	BB	Bb	bb								
	Total	**647**	**1449**	**1163**	**583**	**1428**	**1426**	1.07 [0.82, 1.41] ^c^	1	0.90 [0.64, 1.26] ^c^	1	0.99 [0.75, 1.31] ^c^	1	0.86 [0.72, 1.03] ^c^	0.36
	ES Asians	0	0	0	0	0	0	NA		NA		NA		NA	
	SW Asians	540	935	478	438	726	419	1.09 [0.77, 1.53] ^c^	1	0.88 [0.58, 1.33] ^c^	1	0.99 [0.70, 1.40] ^c^	1	0.82 [0.63, 1.06] ^c^	0.48
	Africans	69	410	633	125	584	929	1.33 [0.96, 1.84]	0.32	1.35 [0.98, 1.86]	0.24	1.34 [0.98, 1.82]	0.28	1.06 [0.91, 1.24]	1
	Americans	0	0	0	0	0	0	NA		NA		NA		NA	
	Europeans	38	104	52	20	118	78	0.47 [0.24, 0.91]	0.08	0.34 [0.07, 1.58] ^c^	0.68	0.41 [0.22, 0.76]	0.02	0.62 [0.24, 1.58] ^c^	1
FokI		FF	Ff	ff	FF	Ff	Ff								
	Total	**2116**	**1433**	**369**	**2160**	**1566**	**337**	0.89 [0.76, 1.05] ^c^	0.64	1.24 [0.91, 1.70] ^c^	0.68	0.94 [0.79, 1.11] ^c^	1	1.36 [1.14, 1.62]	0.0028
	^f^ES Asians	45	106	79	111	167	64	1.52 [0.99, 2.33]	0.20	2.55 [1.55, 4.19]	0.0008	1.81 [1.21, 2.71]	0.016	1.97 [1.32, 2.93]	0.0032
	SW Asians	1025	707	150	708	601	60	0.71 [0.55, 0.93] ^c^	0.04	1.37 [0.74, 2.51]	1	0.77 [0.58, 1.01] ^c^	0.24	1.58 [0.94, 2.65] ^c^	0.32
	Africans	944	509	71	1211	641	95	1.04 [0.90, 1.20]	1	0.96 [0.70, 1.33]	1	1.03 [0.89, 1.18]	1	0.95 [0.69, 1.31]	1
	Americans	44	51	59	95	120	110	0.89 [0.51, 1.54]	1	0.84 [0.35, 1.98]	1	0.92 [0.54, 1.56]	1	1.20 [0.74, 1.94]	1
	Europeans	58	60	10	35	37	8	0.98 [0.54, 1.76] ^d^	1	0.75 [0.27, 2.09] ^d^	1	0.94 [0.53, 1.65] ^d^	1	0.76 [0.29, 2.02] ^d^	1
TaqI		TT	Tt	tt	TT	Tt	tt								
	Total	**2553**	**1842**	**412**	**2864**	**2082**	**471**	1.05 [0.91, 1.20] ^c^	1	1.03 [0.77, 1.37] ^c^	1	1.04 [0.90, 1.21] ^c^	1	0.97 [0.76, 1.24] ^c^	1
	ES Asians	430	42	6	299	42	5	0.79 [0.48, 1.32]	1	1.33 [0.37, 4.76]	1	0.86 [0.53, 1.39]	1	1.37 [0.38, 4.91]	1
	SW Asians	712	735	233	710	592	197	1.23 [0.97, 1.57] ^c^	0.36	1.27 [0.76, 2.10] ^c^	1	1.26 [0.95, 1.67] ^c^	0.44	1.08 [0.73, 1.60] ^c^	1
	Africans	1234	910	155	1554	1272	233	0.89 [0.79, 1.00]	0.2	0.86 [0.59, 1.24] **^c^**	1	0.88 [0.79, 0.98]	0.12	0.89 [0.62, 1.27] **^c^**	1
	Americans	112	38	4	228	89	6	0.92 [0.56, 1.49]	1	1.69 [0.48, 5.96]	1	0.94 [0.58, 1.51]	1	1.56 [0.46, 5.33]	1
	Europeans	65	117	14	73	87	30	1.70 [0.61, 4.74] **^c^**	1	0.47 [0.21, 1.04]	0.24	1.35 [0.87, 2.07]	0.72	0.20 [0.01, 6.66] **^c^**	1

^a^ Dominant model: the combination of variant homozygote genotype and heterozygote genotype versus the wild-type homozygote.

^b^ Recessive model: variant homozygote versus the combination of heterozygote genotype and wild-type homozygote genotype.

^c^ Random-effect model was used because of the *P*-value for the heterogeneity test was <0.05.

^d^ Heterogeneity can not be calculated due to one study.

^e^ *P*-value was adjusted by Bonferroni correction, as multiple comparison was done at four times for each SNPs and in subgroups of different ethnicities.

^f^ Data were available from two Chinese studies.

Abbreviations: CI, confidence interval; ES, East and Southeast; Ht, heterozygote; OR, odds ratio; NA, not available; SNP, single-nucleotide polymorphism; SW, South and West; VDR, vitamin D receptor; VR Homo, variant homozygote; WT Homo, wide-type homozygote.

Of the seventeen studies that were conducted in Asia, two were form China, one was from Cambodia, and the remainder originated from India and Iran. Because the geographical differences might contribute to the genetic diversity, we classified the studies into two groups: East and Southeast Asia (China and Cambodia) and South and West Asia (India and Iran). Additionally, seven African studies were conducted in the sub-Saharan region. As a result, we classified the enrolled studies into five subgroups based on ethnicity and region for the subgroup analysis. The five subgroups included East and Southeast Asians, South and West Asians, Africans, Europeans and Americans.

**Figure 2:**
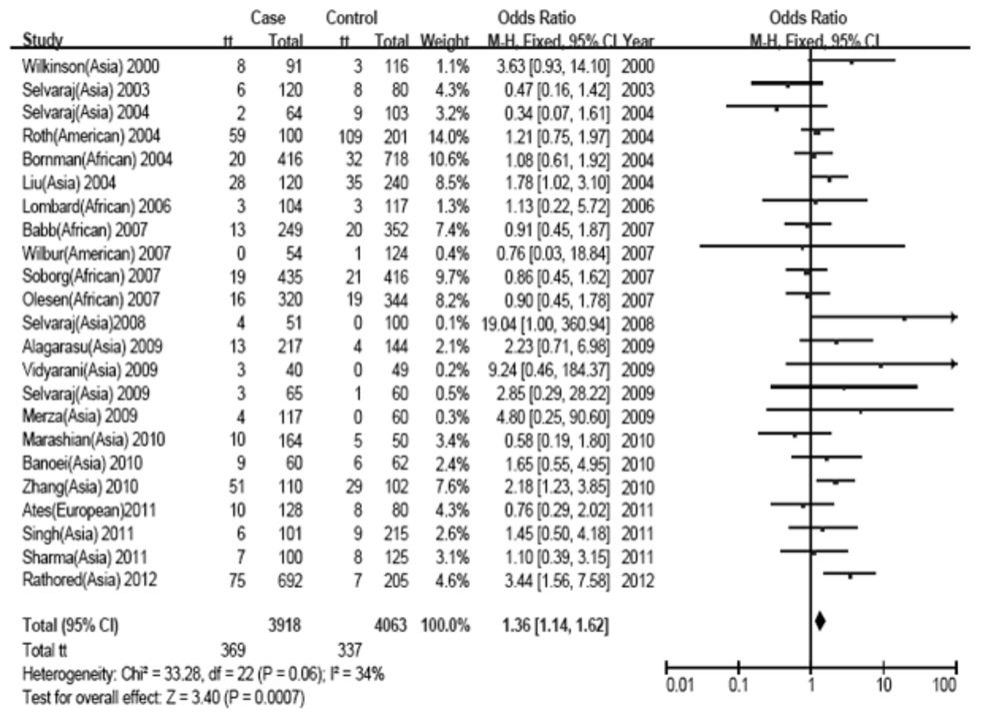
Forest plot of the recessive model of the VDR gene FokI polymorphism on the risk of tuberculosis.

**Figure 3:**
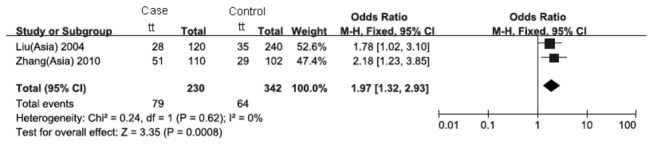
Forest plot of the recessive model of the VDR gene FokI polymorphism on the risk of tuberculosis in Chinese.

**Figure 4:**
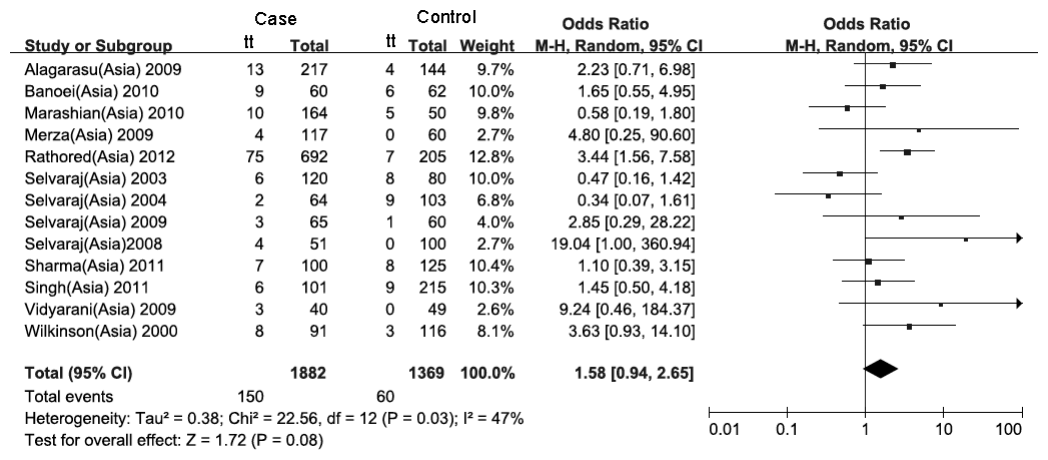
Forest plot of the recessive model of the VDR gene FokI polymorphism on the risk of tuberculosis in Indians and Iranians.

**Figure 5:**
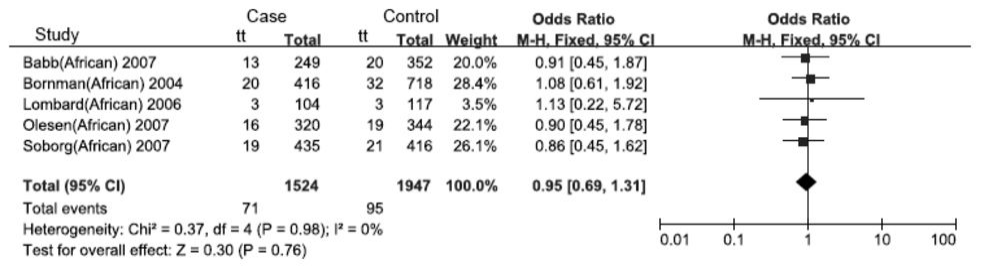
Forest plot of the recessive model of the VDR gene FokI polymorphism on the risk of tuberculosis in Africans.

**Figure 6:**
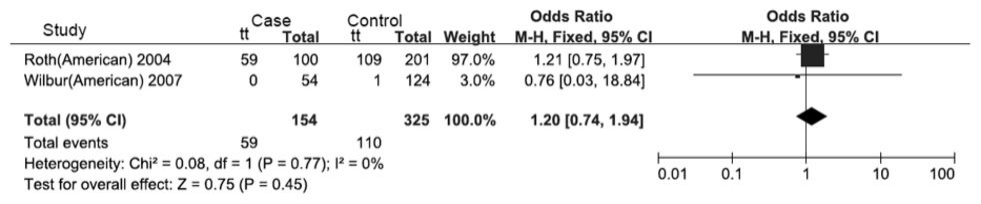
Forest plot of the recessive model of the VDR gene FokI polymorphism on the risk of tuberculosis in Americans.

Of the four types of comparisons, we found that only the variant homozygous genotype of FokI polymorphism was associated with a significantly increased risk of tuberculosis when compared to the heterozygous and wild type homozygous genotypes (OR_*recessive*_=1.36, 95%CI: 1.14-1.62, *P*_*bonferroni*_=0.0028; heterogeneity test: χ^2^=33.28, *P*=0.06) (Figure 2). Furthermore, when stratifying the different ethnicities (there were only two Chinese studies conducted in East and Southeast Asians), a significant association of recessive model was only found in Chinese (OR_*recessive*_=1.97, 95%CI: 1.32-2.93, *P*_*bonferroni*_=0.0032; heterogeneity test: χ^2^=0.24, *P*=0.62) (Figure 3). Also in the same stratification, the variant homozygote of the FokI polymorphism significantly increased the risk of tuberculosis by 1.55 fold when compared to the wild type (OR=2.55, 95%CI: 1.55-4.19, *P*_*bonferroni*_=0.0008; heterogeneity test: χ^2^=0.15, *P*=0.70), and the dominant model of the FokI polymorphism was also significantly increased tuberculosis risk by 0.81 fold (OR_*dominat*_=1.81, 95%CI: 1.21-2.71, *P*_*bonferroni*_=0.016; heterogeneity test: χ^2^=0.13, *P*=0.72). The protective effect of heterozygote of the FokI polymorphism was in marginal significance in South and West Asians (*P*=0.04). The forest plots of the VDR gene FokI polymorphism (recessive model) in South and West Asians, Africans and Americans showed no significant associations (Figure 4, 5 and 6). Because only one study was found for FokI in Europe, that forest plot was not implemented.

The other three SNPs of the VDR gene showed no notable associations with tuberculosis risk in the pooled samples. However, stratification of ethnicities revealed some evidence of tuberculosis risk for the other three SNPs. 

For the BsmI polymorphism in the European population, the variant homozygote and heterozygote genotypes were associated with a significantly decreased risk of tuberculosis when compared to the wild type homozygote (OR_*dominant*_=0.41, 95%CI: 0.22-0.76, *P*_*bonferroni*_=0.02; heterogeneity test: χ^2^=2.59, *P*=0.11). Although the heterozygote of the BsmI polymorphism decreased the risk of tuberculosis by 0.53-fold when compared to the wild type homozygote (OR=0.47, 95%CI: 0.24-0.91; *P*_*bonferroni*_=0.08; heterogeneity test: χ^2^=1.17, *P*=0.28), the Bonferroni correction showed no significance. 

For the TaqI polymorphism in the African population, the variant homozygote and heterozygote genotypes reduced the risk of tuberculosis when compared to the wild type homozygote by marginal significance (OR_*dominant*_=0.88, 95%CI: 0.79-0.98, *P*_*bonferroni*_=0.12; heterogeneity test: χ^2^=8.51, *P*=0.20). Also, the heterozygote of the TaqI polymorphism reduced the risk of tuberculosis by 0.11 fold when compared to the wild type homozygote (OR=0.89, 95%CI: 0.79-1.00; *P*_*bonferroni*_=0.2; heterogeneity test: χ^2^=1.17, *P*=0.28). However, the adjusted *P*-values of the above results showed no significance by Bonferroni correction.

Because only one study was conducted in the European population for the ApaI polymorphism, the heterogeneity and pooled ORs could not be calculated. 

Meta regression was adopted to illustrate the potential reasons for heterogeneity between the studies if the *P*-value of that heterogeneity was less than 0.05. However, the *P*-value of the heterogeneity test for the significant association (recessive model of FokI polymorphism) exceeded 0.05. Therefore, we did not perform further meta-regression.

### Publication bias

**Figure 7:**
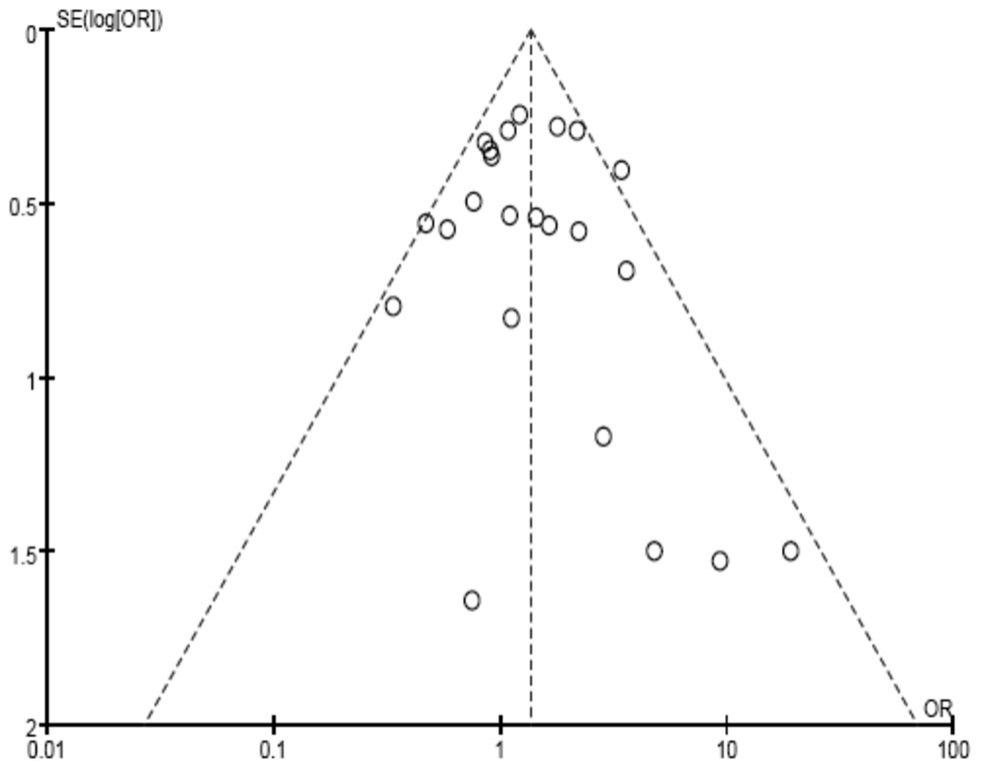
Funnel plot of the recessive model of the VDR gene FokI polymorphism on the risk of tuberculosis, Egger's Test: *t*=0.51, *P*=0.614. log, logarithms; SE, standard error.

In this meta-analysis, a Funnel plot, Egger’s test and Horbold-Egger’s test was used to evaluate the potential publication bias of the enrolled studies. For the significant result of the FokI recessive model, the Egger’s test (*t*=0.51, *P*=0.614) showed no evidence of publication bias. However, the funnel plot of the FokI recessive model suggested some asymmetry that might be induced by small sample sizes (Figure 7). However, the Horbold-Egger’s test supported the result of Egger’s test that suggested that the publication bias induced by small sample sizes was limited (*t*=0.48, *P*=0.633).

## Discussion

In this meta-analysis of the relationship between the VDR gene polymorphisms and the risk of tuberculosis, we found that the FokI SNP in the recessive model (ff vs. Ff/FF) significantly increased the risk of tuberculosis, and the risk effect was found in the Chinese population. The other three SNPs variants were not linked to any significant risk or protective effect on tuberculosis in the total population. However, in the stratification of different ethnicities, we found that the dominant model of BsmI SNP generated a protective effect on tuberculosis in the European population. 

Because the frequency of minor alleles of each SNP of VDR gene was commonly more than 0.01 in the different populations based on the HapMap data (http://hapmap.ncbi.nlm.nih.gov/), the four SNPs are valuable for tuberculosis susceptibility research in different ethnic backgrounds. Genetic alterations of the VDR gene may lead to changes in gene activation or in VDR protein structure, and these changes could affect the cellular function of 1,25-dihydroxyvitamin D3. Additionally, VDR gene polymorphisms could be linked to each other and to other unidentified genes, which could also be important for tuberculosis risk.

The FokI SNP was located within the exon 2 of the VDR gene, and evidence of FokI functionality has already been obtained [41]. Results from transcriptional activation studies in transfected HeLa cells using a reporter construct under the control of a short portion of the rat 24-hydroxylase gene promoter region (-291-+9) containing a vitamin D responsive element (VDRE) suggested that the short 424 amino acid VDR protein variant (corresponding with the C-allele or ‘‘big F’’ allele) would be more active than the long 427 aa variant. Because the luciferase activities of transfected HeLa cells that containing different variants of FokI increased by 19.5- (ff) and 11.2-fold (FF), respectively. Thus, a 1.7-fold relative difference was observed between the two variants. Thus, the f allele of FokI might decrease the activity of the VDR protein and deter the binding of active vitamin D and VDR. Our results on the FokI polymorphism were in accordance with a previously conducted meta-analysis which indicated that the f allele in a recessive model would increase the risk of tuberculosis, and the same effect was found in the Chinese population but not for other ethnicities [12]. The allele frequencies might be different in various ethnicities; therefore, the risk genotype would be changeable in different ethnicities. The risk effect of the f allele of FokI was not found in the African population, which may be explained by the different f allele frequency in this population (0.192), while the f allele frequency of FokI was 0.442 in Asia (http://hapmap.ncbi.nlm.nih.gov/). Also, due to the small sample size, the analysis of the effect of the FokI polymorphism on tuberculosis was underpowered.

The ApaI and BsmI polymorphisms were located in the intron region between exon 8 and exon 9 of the VDR gene. Although the nucleotide changes of the ApaI and BsmI polymorphisms generated no changes in the amino acid or the structure of the expressed VDR protein, they might be in linkage disequilibrium with other functional polymorphisms which regulated VDR gene expression. Earlier studies have provided evidence of differential luciferase activity for the two 3' UTR variants that were linked to the most frequent haplotypes [42]. In this meta-analysis, homozygote for the variant allele of the ApaI polymorphism ad heterozygote for the BsmI polymorphism both appeared to have a protective role on tuberculosis development in European population. However, due to the small sample size of the enrolled studies, this result needed to be validated in a larger European population.

The allele changes of the TaqI SNP would not generate an amino acid transformation. The t allele of the TaqI SNP seemed to be protective for tuberculosis in the African population, although the results were only marginally significant. This different might be explained by different ethnic backgrounds. The t allele frequency (HapMap data) of TaqI was 0.012 in the Chinese population and 0.118 in the Japanese population, 0.288 in the African population and 0.438 in the European population. Meanwhile, It has been suggested that the mRNA coded from the TaqI t allele of the VDR gene would be more stable than the mRNA from the T allele of the VDR gene [43], and a previous study revealed that the TaqI SNP in exon 9 near 3′ UTR was in linkage disequilibrium with the ApaI and BsmI polymorphisms [44], which may explain the same protective effect by the two variant alleles of the TaqI and BsmI polymorphisms on tuberculosis. 

Finally, some limitations of this study need to be addressed. First, HIV status might influence tuberculosis incidence; so, the stratification of HIV status would further reveal the relationship between VDR gene SNPs and tuberculosis. However, HIV status was not reported in one-third of the enrolled studies. Therefore, it was not possible to apply stratification according to HIV status. Second, subgroup analysis with a small number of studies would reduce the statistical power of the analysis. Third, the main method for tuberculosis diagnosis was based on bacteriology. However, these bacteriological methods could not distinguish between *Mycobacterium tuberculosis* and *non-tuberculosis Mycobacterium* (*non-tuberculosis Mycobacterium* constituted a small portion of the clinical cases), which might exert a confounding effect.

In conclusion, this meta-analysis study evaluated the relationship between the VDR gene hot spot SNPs and the risk of tuberculosis in twenty-nine case-control studies, and provided evidence that the FokI SNP might increase the risk of tuberculosis in a recessive model. Thus, host genetic susceptibility might be involved in tuberculosis development. However, due to the small sample sizes, conclusions could not be drawn regarding the putative protective effect of the BsmI variant allele in the European population. Studies with larger sample sizes studies are warranted to be undertaken.

## Supporting Information

Checklist S1 **PRISMA Checklist.**
(DOC)

Figure S1 **Forest plot of the dominant model of VDR gene ApaI polymorphism.**
(PDF)

Figure S2 **Forest plot of the recessive model of VDR gene ApaI polymorphism.**
(PDF)

Figure S3 **Forest plot of the dominant model of VDR gene BsmI polymorphism.**
(PDF)

Figure S4 **Forest plot of the recessive model of VDR gene BsmIpolymorphism.**
(PDF)

Figure S5 **Forest plot of the dominant model of VDR gene FokIpolymorphism.**
(PDF)

Figure S6 **Forest plot of the dominant model of VDR gene TaqI polymorphism.**
(PDF)

Figure S7 **Forest plot of the recessive model of VDR gene TaqI polymorphism.**
(PDF)
