# Supplementary figures and images for: Vitamin D Receptor Gene Polymorphisms on the Risk of Tuberculosis, a Meta-Analysis of 29 Case-Control Studies
Source: PLoS One. 2013 Dec 13;8(12):e83843. doi: 10.1371/journal.pone.0083843 (PMC3862802; doi:10.1371/journal.pone.0083843)

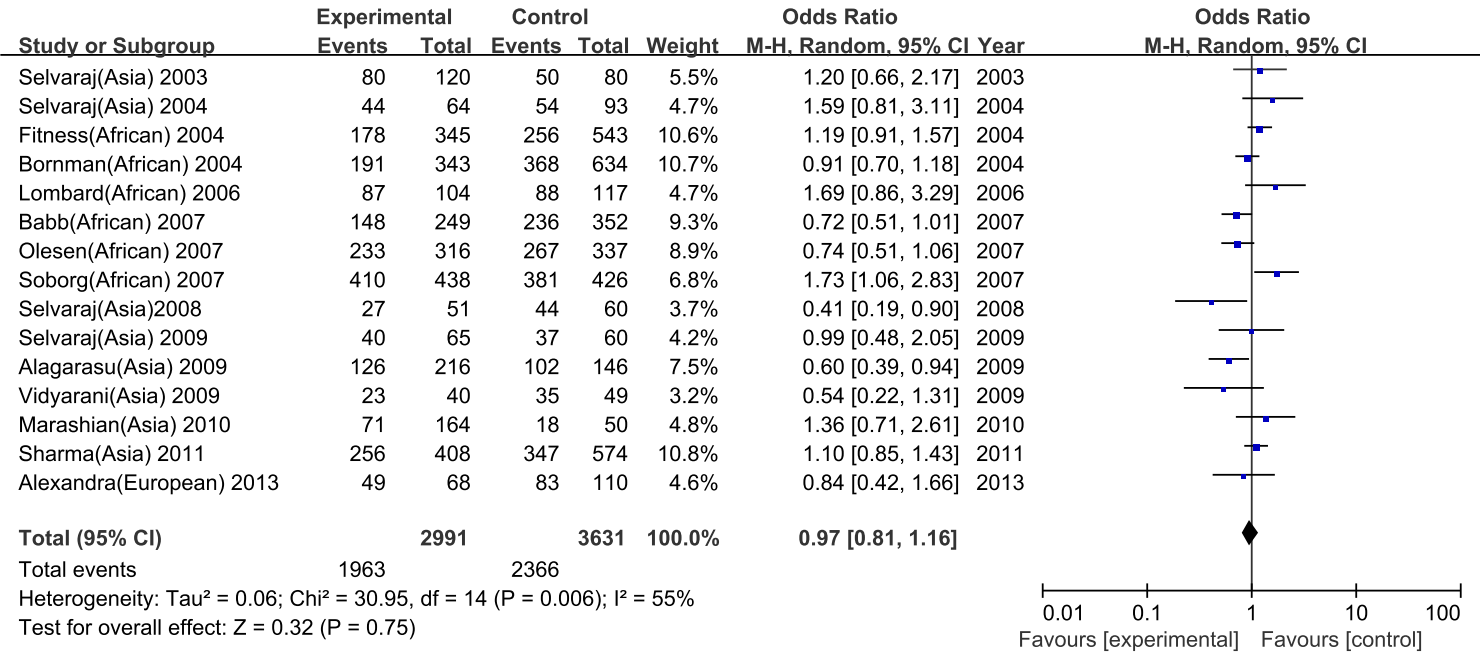

Supplement: Figure S1 — Forest plot of the dominant model of VDR gene ApaI polymorphism. (PDF) [file pone.0083843.s002.pdf]

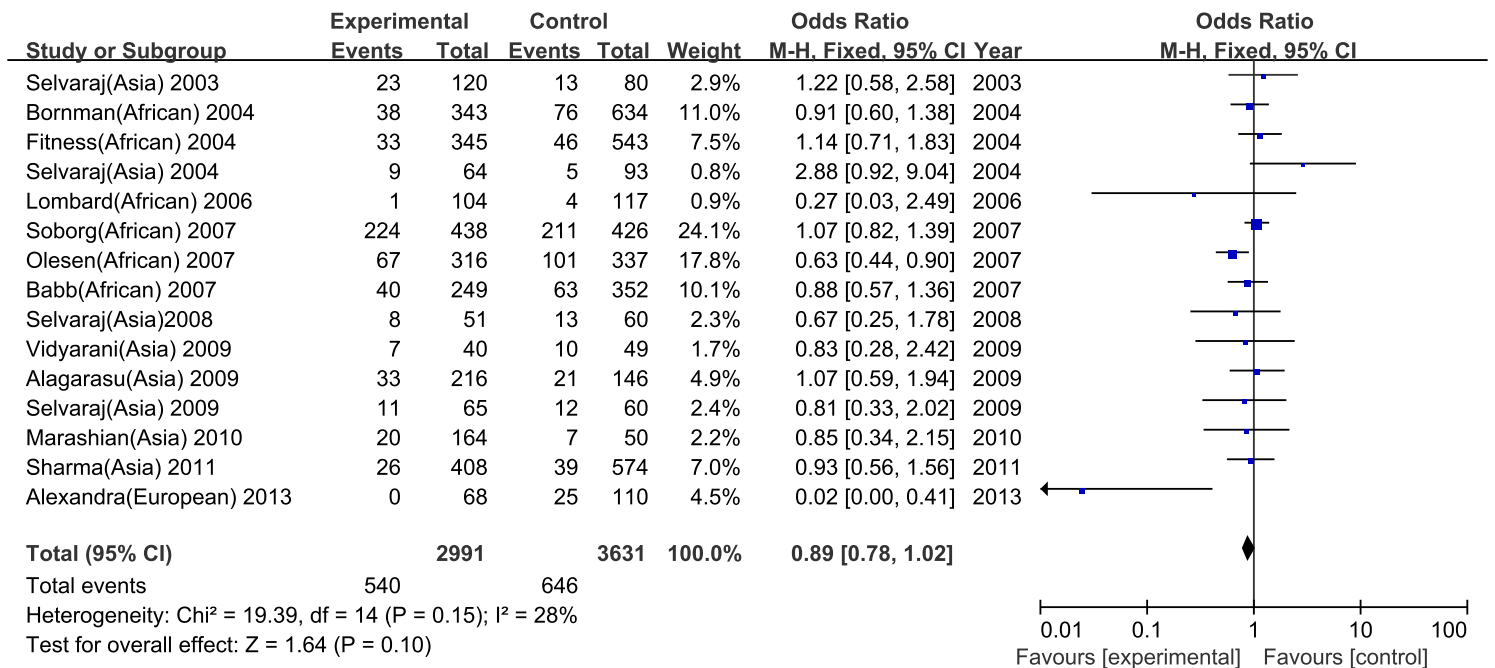

Supplement: Figure S2 — Forest plot of the recessive model of VDR gene ApaI polymorphism. (PDF) [file pone.0083843.s003.pdf]

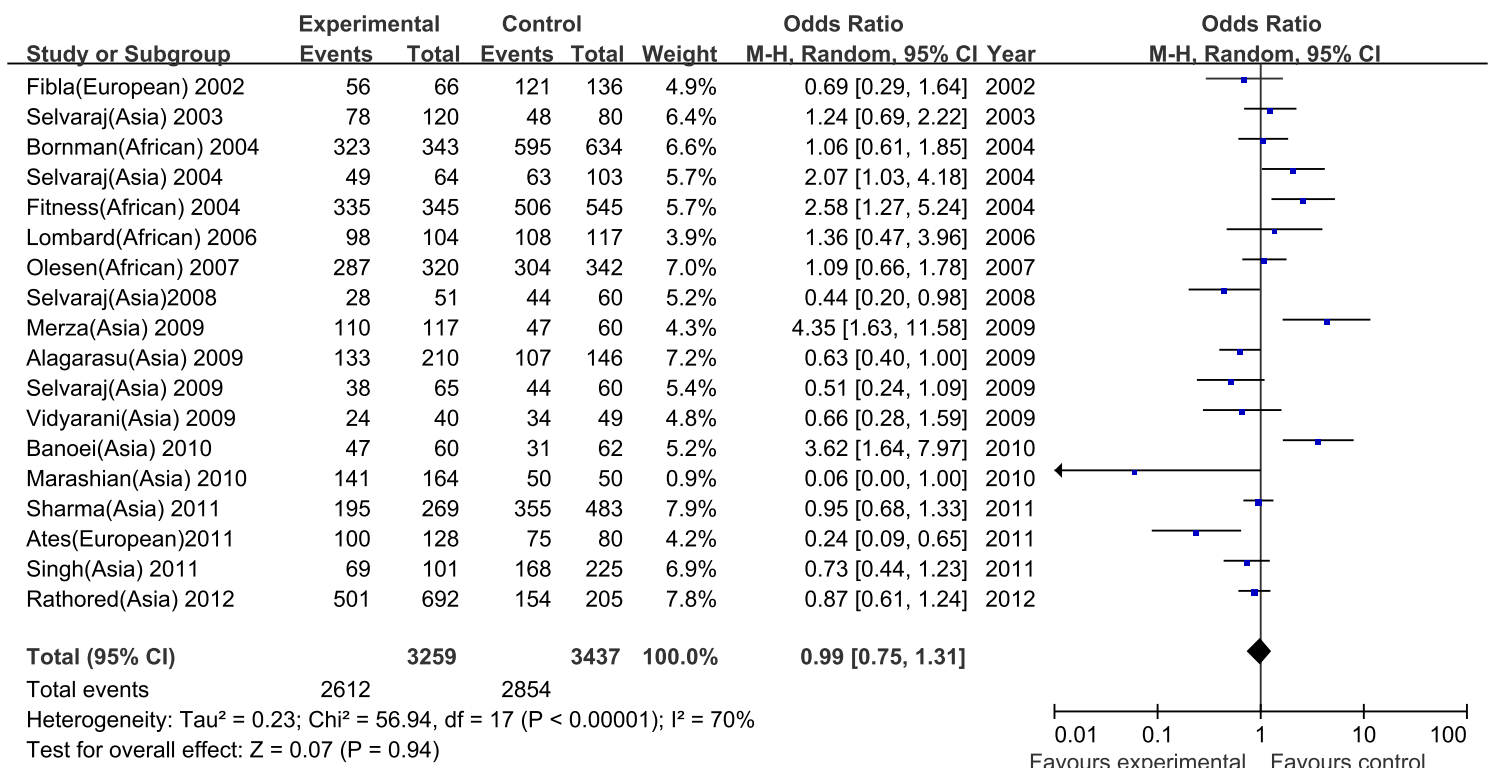

Supplement: Figure S3 — Forest plot of the dominant model of VDR gene BsmI polymorphism. (PDF) [file pone.0083843.s004.pdf]

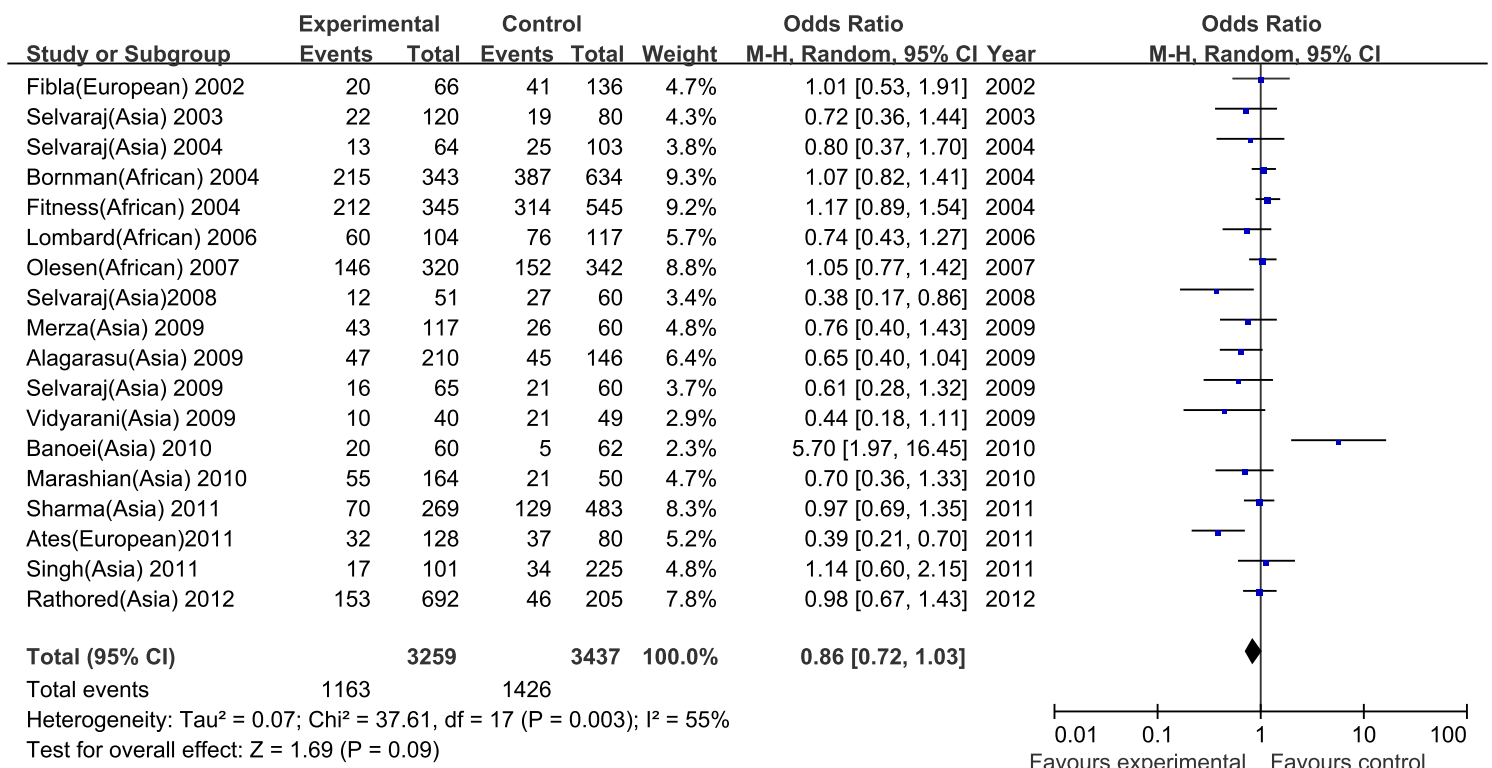

Supplement: Figure S4 — Forest plot of the recessive model of VDR gene BsmIpolymorphism. (PDF) [file pone.0083843.s005.pdf]

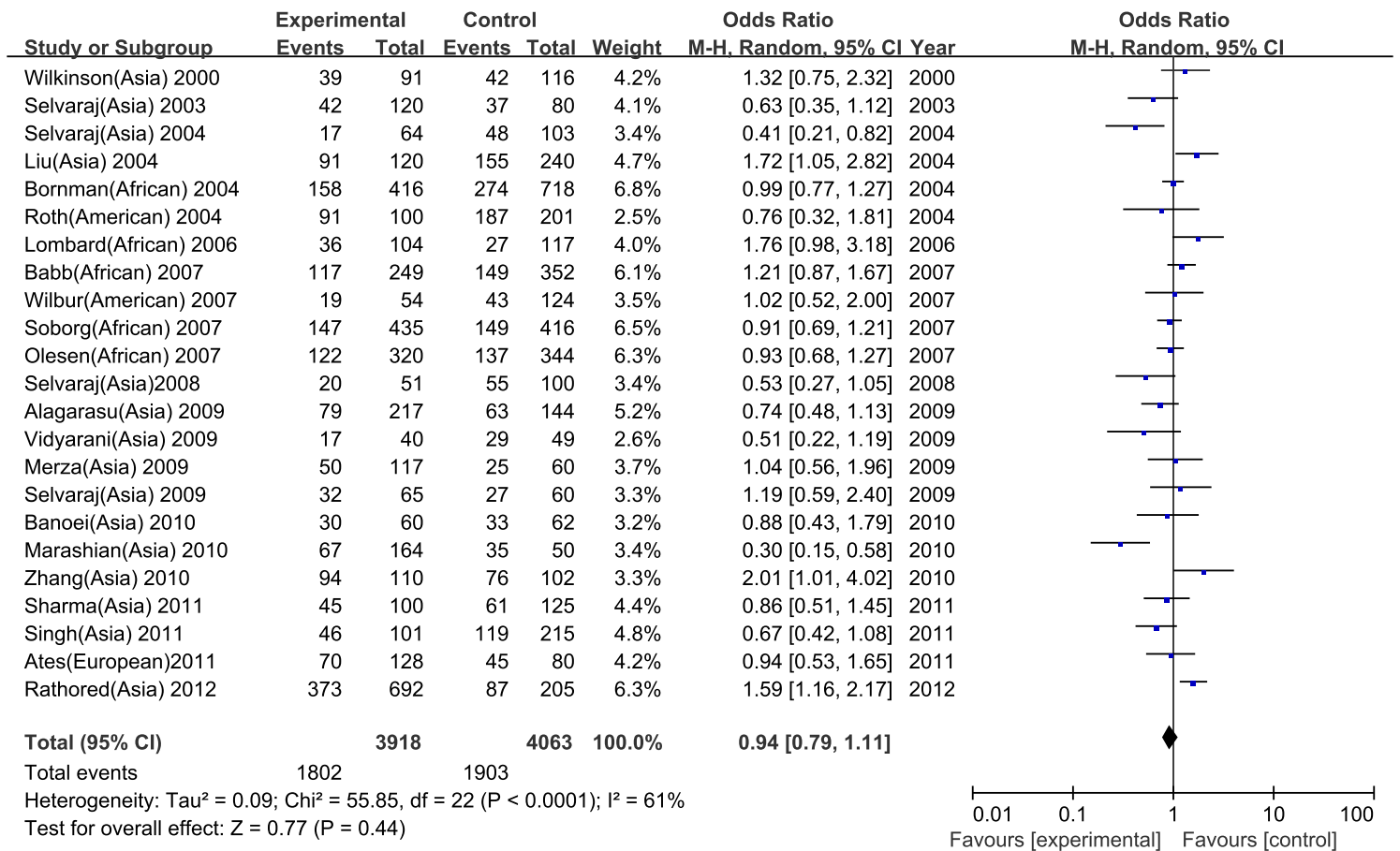

Supplement: Figure S5 — Forest plot of the dominant model of VDR gene FokIpolymorphism. (PDF) [file pone.0083843.s006.pdf]

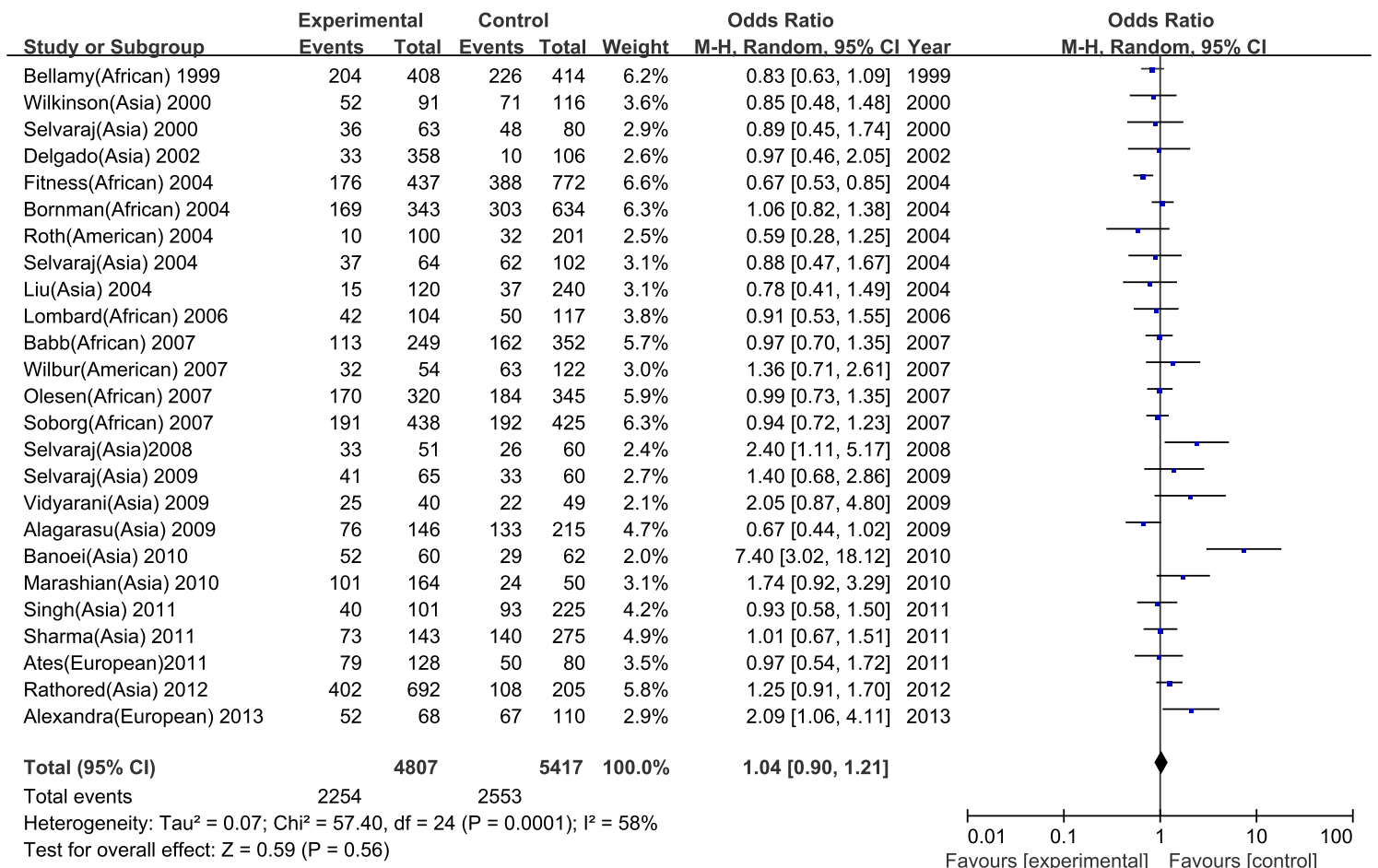

Supplement: Figure S6 — Forest plot of the dominant model of VDR gene TaqI polymorphism. (PDF) [file pone.0083843.s007.pdf]

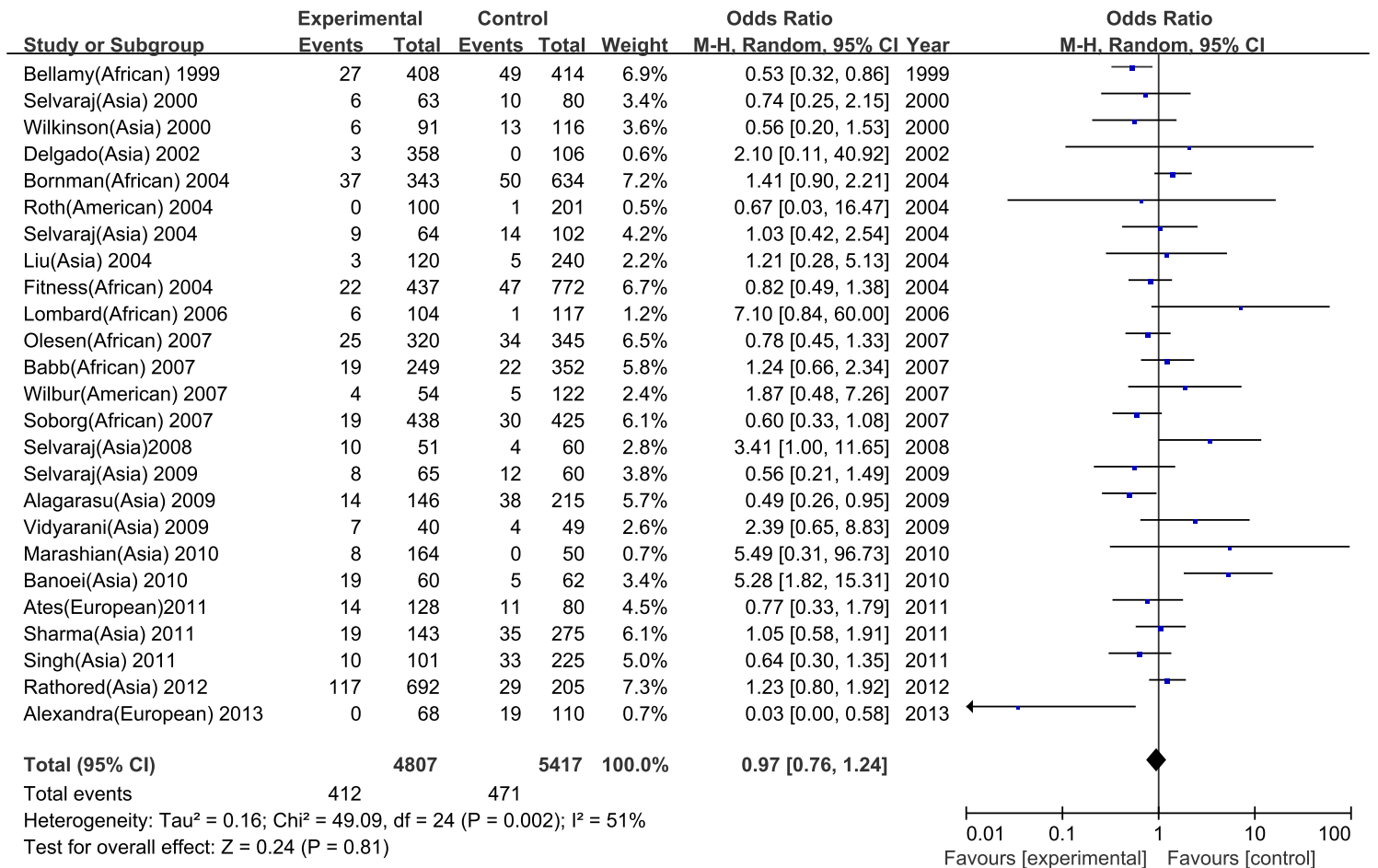

Supplement: Figure S7 — Forest plot of the recessive model of VDR gene TaqI polymorphism. (PDF) [file pone.0083843.s008.pdf]
